# Enhanced Effect of *β*-Lactoglobulin Immunization in Mice with Mild Intestinal Deterioration Caused by Low-Dose Dextran Sulphate Sodium: A New Experimental Approach to Allergy Studies

**DOI:** 10.3390/nu16203430

**Published:** 2024-10-10

**Authors:** Dagmara Złotkowska, Lidia Hanna Markiewicz, Anna Maria Ogrodowczyk, Barbara Wróblewska, Ewa Wasilewska

**Affiliations:** Department of Immunology and Food Microbiology, Institute of Animal Reproduction and Food Research of the Polish Academy of Sciences, Tuwima 10 Str., 10-748 Olsztyn, Poland

**Keywords:** cow’s milk protein allergy (CMPA), mouse model, DSS-induced early intestinal barrier dysfunction, humoral and cellular response, immune mediators, microbiota activity

## Abstract

Background/Objectives: Cow’s milk allergy is one of the most common food allergies in children, and its pathomechanism is still under investigation. Recently, an increasing number of studies have linked food allergy to intestinal barrier dysfunction. The present study aimed to investigate changes in the intestinal microenvironment during the development of *β*-lactoglobulin (*β*-lg) allergy under conditions of early intestinal dysfunction. Methods: BALB/c mice received intraperitoneal *β*-lg with Freund’s adjuvant, followed by oral *β*-lg while receiving dextran sulphate sodium salt (DSS) in their drinking water (0.2% *w*/*v*). The immunized group without DSS and the groups receiving saline, oral *β*-lg, or DSS served as controls. Results: The study showed that the immunization effect was greater in mice with mild intestinal barrier dysfunction. Although DSS did not affect the mice’s humoral response to *β*-lg, in combination with *β*-lg, it significantly altered their cellular response, affecting the induction and distribution of T cells in the inductive and peripheral tissues and the activation of immune mediators. Administration of *β*-lg to sensitized mice receiving DSS increased disease activity index (DAI) scores and pro-inflammatory cytokine activity, altered the distribution of claudins and zonulin 1 (ZO-1) in the colonic tissue, and negatively affected the balance and activity of the gut microbiota. Conclusions: The research model used appears attractive for studying food allergen sensitization, particularly in relation to the initial events leading to mucosal inflammation and the development of food hypersensitivity.

## 1. Introduction

Food hypersensitivity is a term that encompasses a variety of ailments caused by the body’s abnormal response to foods that are harmless to healthy individuals. Epidemiological data indicate that it is a growing public health problem. In particular, there is a steady and rapid increase in the incidence and severity of symptoms of allergic diseases in both children and adults. Particularly allergenic are proteins from cow’s milk, egg, nuts, wheat, and soya, as well as mollusks and shellfish. There is no effective treatment for food allergy other than a properly selected elimination diet, so research into the topic is still of the utmost importance.

*β*-lactoglobulin is a major allergenic milk protein (Bos d 5) accounting for approximately 50% of whey protein and 10% of the total protein content of milk. Sensitization to Bos d 5 occurs in 80% of patients with CMPA (cow’s milk protein allergy), and cross-reactivity of the protein with other food allergens is possible. The protein contains several epitopes located in different parts of the tertiary structure that have the ability to bind to specific IgE. In the gut, the ingested allergen is processed and absorbed into the epithelium, and allergen-derived peptides are present on the surface of antigen-presenting cells (APCs). Directed by cytokines or thymic stromal lymphopoietin (TSLP) or damage-associated molecular patterns (DAMPs) activated by these APCs, allergen-specific T cells differentiate into T helper 2 (Th2) cells and induce local activation of innate type II lymphocytes (ILC2s). Activated ILC2 cells provide IL-13 and IL-4, which drive Th2 cell activation and differentiation, thus allowing activation, differentiation, and isotype switching of allergen-specific B cells into IgE-producing plasma cells. Allergen-specific IgE antibodies are then produced, which bind to the high-affinity IgE receptor FcεRI on the surface of mast cells and basophils, sensitizing them to the allergen. Upon a second exposure to the allergen, a process of mast cell and eosinophil degranulation occurs, causing allergic inflammation and associated symptoms [1,2]. Less obvious and recognized pathways of sensitization have also been proposed [3].

The pathogenesis of allergy remains unclear, but there is a growing belief that modern lifestyles and environmental changes are the main triggers. Food allergy is increasingly described as being associated with an apparent defect in intestinal barrier function [4,5]. Patients with food allergy have been shown to have defects in the epithelial barrier and in the induction of regulatory T lymphocytes (Tregs) and the production of neutralizing, allergen-specific IgA antibodies, or changes in the composition of the microbiota [1,6,7,8]. Increased intestinal permeability allows allergens to infiltrate the intestinal barrier and stimulate the submucosal immune system, and the cytokines and inflammatory mediators released in this process increase barrier breakdown and permeability. Despite this knowledge and continued intensive research, it is difficult to understand the exact mechanism that initiates the development of allergy and to translate this knowledge into therapeutic approaches, partly due to the lack of well-matched experimental models. Pre-clinical mouse models are essential for allergy research and the exploration of new therapeutic approaches. As there are no naturally occurring allergies in mice, it is difficult to develop a methodology that fully matches human pathology [2]. Epidemiological studies have shown that bowel disease increases the prevalence of cow’s milk allergy [9,10]. More recently, Chen et al. [11] described a model of food allergy in mice with dextran sulphate sodium (DSS)-induced colitis, but the model is based on the study of allergy in mice with acute inflammation. Allergen sensitization and disease initiation begin at barrier sites, such as the cohesive epithelium lining the dynamic immune system. We hypothesized that even a small but prolonged breach of this barrier could lead to overactivity of gut-associated lymphoid tissue (GULT) and the development of allergy. This could be due to uncontrolled infiltration of antigens from the gut lumen. Our aim was to investigate whether mild intestinal deterioration would affect the sensitization of mice to allergenic milk proteins, such as *β*-lactoglobulin. We assumed that a low dose of a pro-inflammatory agent, such as DSS, would increase the hyper-reactivity of the immune response to the milk allergen due to the pro-inflammatory activity of this agent and the deleterious effect on the epithelium.

## 2. Materials and Methods

### 2.1. Animals

Specific-pathogen-free BALB/cCmdb mice (8-week-old females) weighing 20 ± 2 g were used in this study. The mice were purchased from the Experimental Medicine Centre in Białystok, Poland. During the experiments, they were housed in a pathogen-free barrier facility and maintained in accordance with relevant guidelines and regulations. Mice were fed a maintenance pathogen-free diet for rats and mice (#1320, Altromin Spezialfutter GmbH, Lage, Germany) free from milk proteins. Water and food were provided ad libitum.

### 2.2. Experimental Design

**Figure 1:**
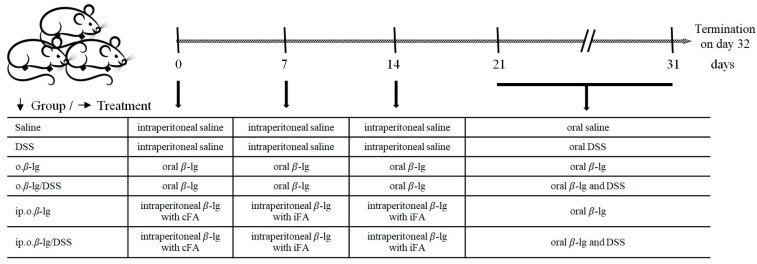
Experimental design. Abbreviations used: *β*-lg—*β*-lactoglobulin; cFA—complete Freund adjuvant; iFA—incomplete Freund adjuvant.

For the experiment, the animals were randomly assigned to 6 groups (n = 10/group): saline—control group, administered saline; DSS—administered DSS (orally); o.*β*-lg—administered *β*-lg orally; o.*β*-lg/DSS—administered *β*-lg and DSS orally; ip.o.*β*-lg—injected intraperitoneally and administered orally with *β*-lg; ip.o.*β*-lg/DSS—injected intraperitoneally with *β*-lg and administered orally with *β*-lg and DSS (Figure 1). Intraperitoneal immunization (ip.) was conducted with 300 µg of *β*-lg with Freund’s adjuvant on days 0, 7, and 14. Oral antigen delivery was conducted with 200 µg of *β*-lg administered intragastrically from day 21 to day 31. Dextran sulphate sodium salt (DSS; molecular weight = 36,000–40,000 Da; Sigma-Aldrich, Steinheim, Germany) was administered in drinking water (0.2%) ad libitum from day 21 to day 31. Across all experimental steps, the control group received saline.

Mice were monitored daily for body weight (BW), stool consistency, and appearance, and the disease activity index (DAI) scores were calculated as described in Section 2.3. Mice were terminated on day 32 through carbon dioxide inhalation, after which the abdominal skin was sprayed with 70% ethanol and blood was drawn through cardiac puncture. Subsequently, spleens (SPLs), mesenteric lymph nodes (MLNs), head and neck lymph nodes (HNLNs), Peyer’s patches (PPs), small and large intestine tissues, and cecal contents were collected for lymphocyte isolation or genetic or microbiological testing. Samples were analyzed fresh or frozen in liquid nitrogen and stored at −80 °C until analysis. All animal care and procedures conformed to the tenets of the Declaration of Helsinki and were approved by the Local Ethics Committee for Animal Experiments in Olsztyn (15/2021).

### 2.3. DAI and Body Weight

The DAI score was computed as the combined scores of the scoring system (sum) over the evaluation period, comprising the following: percentage of body weight (BW) loss (increase = 0, 0–5% weight loss = 1, >5% = 2), stool consistency (normal pellets = 0, semi-loose = 1, loose = 2), and appearance (lively/normal = 0, ruffled fur and hunched back = 1, lethargic = 2). Evaluation was conducted at the time of DSS administration, i.e., from day 21 to day 31 of the experiment. The percentage of BW loss was calculated for days 21–31 with reference to the baseline value obtained immediately before oral *β*-lg and DSS administration (day 21).

### 2.4. Antibody Measurements

The total IgE was determined through sandwich ELISA using a mouse IgE ELISA kit (157718; Abcam, Cambridge, UK) according to the manufacturer’s protocol. Serum IgA and IgG and fecal IgA specific for *β*-lg were measured through indirect ELISA. Briefly, 96-well plates were coated with an antigen solution (10 µg/mL) and incubated at 37 °C for 1.5 h. Non-specific protein binding sites were then blocked with 1.5% gelatin diluted in PBS (10 mM, pH 7.4) under the same conditions. After blocking, the plates were washed three times with PBST (PBS + 0.05% Tween 20), and serial dilutions of serum or feces extracts (100 µL) were added and incubated for 1.5 h at 37 °C. The plates were then washed and incubated with anti-mouse peroxidase-conjugated secondary antibodies (Goat anti-mouse IgG and Goat anti-Mouse IgA; Sigma-Aldrich, Saint Louis, MO, USA) for 1 h at 37 °C. After further washing, peroxidase substrate (ABTS; Millipore, Temecula, CA, USA) was added, and after 1 h of incubation at room temperature (RT), the absorbance was measured at 405 nm on a Jupiter UVM spectrophotometer (ASYS-Hitech GmbH, Eugendorf, Austria). Endpoint titers (Epts) were expressed as the reciprocal dilution of the last sample dilution of 0.1 OD over the negative control.

### 2.5. Lymphocyte Isolation and Phenotyping

Lymphocytes were isolated from the PPs, MLNs, SPLs, HNLNs, and blood. Tissues were homogenized in incomplete medium (IM: RPMI-1640 supplemented with 1 mM of HEPES and 10 U/mL of penicillin–streptomycin solution (Sigma-Aldrich, St. Louis, MO, USA) using a glass homogenizer. The resulting suspensions were filtered through an 80 µm mesh nylon membrane and centrifuged at 400× *g* for 10 min at 10 °C. The cell pellets were then resuspended in IM and counted through the trypan blue exclusion method. Before counting, red blood cells were lysed with Red Cells Lysis Buffer (Roche Diagnostics GmbH, Mannheim, Germany). Peripheral blood mononuclear cells (PBMCs) were isolated through a density gradient technique. Anticoagulated blood was subjected to Histopaque-1077 density gradient centrifugation (Sigma-Aldrich, H8889), and cells were collected from the interface, washed with IM medium, and counted.

For phenotyping, lymphocytes were washed twice with FACS buffer (10 mM of PBS supplemented with 2% fetal bovine serum) and stained for cellular markers using a cocktail of anti-mouse BD Pharmingen antibodies conjugated to PerCP-Cy 5—CD4, PE—CD25, and APC-Cy7—CD8 (BD Biosciences, San Jose, CA, USA). After incubation (on ice, 15 min), cells were washed with FACS buffer and fixed and permeabilized with BD Cytofix/Cytoperm Solution (at room temperature, 30 min). Cells were then washed in FACS buffer and intracellularly stained with AF-488 anti-mouse FoxP3 or APC anti-mouse IL-10 (at RT, 30 min) (BD Biosciences). Finally, cells were washed and fixed in 250 µL of 2% PFA and then analyzed using a BD LSRFortessa cell analyzer (BD Biosciences). All incubations were performed in the dark. A minimum of 50,000 events were collected for each sample. Data were analyzed using FlowJo software v. 10.10.0 (Tree Star Inc., Ashland, OR, USA). 

### 2.6. Bacterial DNA Extraction and Cecal Microbiota Enumeration

DNA from cecal contents was isolated using the GeneMATRIX Stool DNA Purification Kit (E3575, EURx, Gdansk, Poland) according to the manufacturer’s instructions. Isolation was performed using a bead-beating method and a Gyrator UNIPRED 3D vortex (UniEquip, Planegg, Germany). The extracted DNA was stored at −20 °C until analysis. The Real-Time quantitative PCR technique was used to quantify *Bifidobacterium* and *Lactobacillus* populations and the total bacteria number (TBN). The detailed methodology, including the strains used for standard curve construction and amplification conditions, has been described previously [12]. The primers used for qPCR are listed in Table 1. Amplification was performed on a Quant Studio 6 Flex system (Thermo Fisher Scientific Inc., Waltham, MA, USA).

### 2.7. Short-Chain Fatty Acid (SCFA) Determination

SCFA analysis was performed on an Agilent 7890A gas chromatography system coupled to a flame ionization detector and a 7683B autoinjector (Agilent Technologies, Santa Clara, CA, USA). Cecal contents preserved in 0.1% trifluoroacetic acid (1:1, vol/vol), frozen in liquid nitrogen, and stored at −80 °C were used for analysis. The SCFAs were separated on an SGE BP21 capillary column (30 m × 0.53 mm; film thickness = 0.5; SGE Analytical Science, Melbourne, Australia). Helium was supplied as the carrier gas at a flow rate of 5 mL/min. The initial oven temperature was 85 °C, and it was then increased to 180 °C at 6 °C/min and held for 5 min. The temperatures of the flame ionization detector and the injection port were 290 °C and 250 °C, respectively. Samples (0.5 μL of clear supernatant) were injected in a split mode at a ratio of 10:1. The content of each compound was measured using external standards of acetic, propionic, butyric, valeric, isovaleric, and isobutyric acid (Sigma-Aldrich, Saint Louis, MO, USA).

### 2.8. Total RNA Extraction, Reverse Transcription, and Gene Expression Analysis

Tissues, approximately 1 cm each from the proximal part of the colon and from the small intestine (jejunum), were snap-frozen in liquid nitrogen and then stored at −80 °C until analysis. Isolation of the total RNA was performed as described by Świątecka et al. [13] with some modifications. Briefly, approximately 50 mg of intestinal tissue was placed in a screw-cap tube containing zirconia beads (Blirt S.A., Gdansk, Poland) and 200 µL of RL buffer (Universal RNA Purification Kit; EURx, Gdansk, Poland) homogenized in a FastPrep-24 homogenizer (MP Biomedicals, Irvine, CA, USA) using the mouse intestine program and centrifuged (11,000× *g*, 2 min, 4 °C). The supernatant obtained was used for RNA extraction using the Universal RNA Purification Kit (EURx, Gdańsk, Poland) according to the manufacturer’s instructions. The concentration and purity of the RNA were assessed spectrophotometrically (NanoDrop; Thermo Fisher Scientific Inc.). Two hundred and fifty nanograms of total RNA were reverse-transcribed (TRANSCRIPTME kit, Blirt, Gdańsk, Poland), and the cDNA obtained was used for real-time PCRs.

Real-time PCR was performed using the Quant Studio 6 Flex system (Thermo Fisher Scientific Inc.) in a total volume of 10 µL containing 5 µL of the PowerUp reagent (Thermo Fisher Scientific Inc.), 1 µL of 2× diluted cDNA, 0.4 µL of each primer, and 3.4 µL water. The expression of interleukins (IL-1β, IL-4, IL-6, and IL-8), tumor necrosis factor alpha (TNF-α), interferon gamma (IFN-γ), transforming growth factor beta (TGF-*β*), toll-like receptors (TLR-2 and TLR-4), claudins (CLDN-2 and CLDN-12), occludin (OCC), and zonulin 1 (ZO-1) was assessed. Expression was normalized to the *β*-actin (ACT) gene using the Relative Quantification application available on the Thermo Fisher Cloud App. The primers used are listed in Table 1. A melting curve was performed to confirm the specificity of the amplicons.

**Table 1:** Primers used in the study.

Target	Forward	Reverse	References
Bacterial primers:			
Eubacteria	gtgstgcayggyygtcgtca	acgtcrtccmcnccttcctc	[14]
*Bifidobacterium*	tcgcgtc(c/t)ggtgtgaaag	ccacatccagc(a/g)tccac	[15]
*Lactobacillus*	agcagtagggaatcttcca	caccgctacacatggag	[16,17]
Mouse primers:			
Interleukin 1β	ttgacggaccccaaaagatg	agaaggtgctcatgtcctcat	[18]
Interleukin 4	gcctgggtcaagctgactac	atgtacgatgtcgccactcc	[12]
Interleukin 6	gacaaagccagagtccttcagagag	ctaggtttgccgagtagatctc	[19]
Interleukin 8	cacctcaagaacatccagagct	caagcagaactgaactaccatcg	[20]
Interferon gamma (IFN-γ)	cgctacacactgcatcttgg	tccttttgccagttcctcca	[21]
Tumor necrosis factor alpha (TNF-α)	ttcctgcaccctctgtctttc	cagttctatggcccagaccc	[13]
Transforming growth factor beta (TGF-*β*)	agacggaatacagggctttcgattca	cttgggcttgcgacccacgtagta	[22]
Toll-like receptor 2 (TLR-2)	gctagcctgccttgtttctc	ggctttttgttgccaaggct	[13]
Toll-like receptor 4 (TLR-4)	gacgctcatgtgagtgagtgta	agagatcacggaccaaggga	[13]
Claudin 2 (CLA2)	ggctgttaggcacatccat	tggcaccaacataggaactc	[23]
Claudin 12 (CLA12)	gtcctctcctttctggcaac	atgtcgatttcaatggcaga	[23]
Occludin (OCC)	gctgtgatgtgtgtgagctg	gacggtctacctggaggaac	[23]
Zonula occludens (ZO-1)	aggacaccaaagcatgtgag	ggcattcctgctggttaca	[23]
*β*-actin (ACT)	ggactcctatgtgggtgacgagg	gggagagcatagccctcgtagat	[24]

### 2.9. Protein Extraction and Western Blotting

Colon and small intestine tissues were homogenized in the FastPrep-24 instrument (the mouse intestine mode) in the presence of RIPA buffer (Sigma-Aldrich, Saint Louis, MO, USA) containing a protease inhibitor cocktail (SIGMAFAST™, S8820, Sigma-Alrdich). The samples were then centrifuged (14,000× *g*, 15 min, 4 °C) and the supernatants were collected and analyzed for protein concentration using the Bradford method. Twenty micrograms of proteins were separated through SDS-PAGE and blotted onto a PVDF membrane. The membranes were then probed with primary antibodies (mouse monoclonal anti-GAPDH, ab9484, Abcam, Cambridge, UK; goat polyclonal anti-ZO-1, AB0054, Sicgen Antibodies, Penela, Portugal; rabbit polyclonal anti-claudin 2, TA347352, OriGene Technologies, Rockville, MD, USA; rabbit polyclonal anti-claudin-12, 38-8200, Thermo Fisher Scientific Inc.) followed by the labeled secondary antibodies (all from Thermo Fisher Scientific Inc.: Alexa Fluor 680 donkey anti-goat IgG, A-21084; Alexa Fluor Plus 680 goat anti-mouse IgG, A32729; Alexa Fluor Plus 800 goat anti-rabbit IgG, A32735) to allow for simultaneous staining of the tested proteins, as previously described [25]. Images of the membranes were acquired using the ChemiDoc Imaging System (Bio-Rad, Hercules, CA, USA) and analyzed using Image Lab v. 6.0 software (Bio-Rad) [26].

### 2.10. Measurements of Cytokines Through Flow Cytometry

Freshly isolated and washed MLN lymphocytes were cultured in CM medium (RPMI 1640 medium supplemented with 1 mM of sodium pyruvate, 1 mM of NEAA, 10 U/mL of penicillin–streptomycin, 10 mM of HEPES, and 10% fetal bovine serum) in 96-well cell culture plates for 4 d at a total volume of 200 μL per well (2.5 × 10^6^ cells/mL). Ex vivo, during culture, lymphocytes were stimulated or not (control) with 100 µg/mL of β-lg, and then the supernatants were collected (through centrifugation at 16,900× *g*, 10 min, 4 °C) and stored at −80 °C until analysis. Concentrations of the cytokines (IL-2, IL-4, IL-6, IFN-γ, TNF-α, IL-17A, and IL-10) in the supernatants were measured using the mouse Th1/Th2/Th17 CBA kit (cat. no. 560485, BD Biosciences, San Jose, CA, USA). Protocols were performed according to the manufacturer’s instructions. Briefly, 50 μL of sample was mixed with 50 μL of the mixed capture beads and 50 μL of mouse detection reagent. After incubation at RT for 2 h in the dark, the samples were washed and resuspended in 300 μL of wash buffer and acquired using a BD LSRFortessa cytometer (BD Biosciences). A minimum of 10,000 events were collected for each sample. Data were analyzed using FCAP Array v. 3.0 software (BD Biosciences).

### 2.11. Statistical Analysis

Data are expressed as means ± SD. ANOVA and Tukey’s post hoc tests were used to evaluate differences between groups for parametric data, whereas the Kruskal–Wallis test was used for nonparametric data. The results were considered significant if *p* < 0.05. The analysis was performed using GraphPad Prism (v. 10.2.0 for Windows; GraphPad Software, San Diego, CA, USA).

## 3. Results and Discussion

There is increasing evidence that patients with food allergy have a disturbed intestinal barrier, but the effect of this disorder in the development of allergy is poorly understood [27,28,29]. In the absence of suitable research models, we investigated whether mild intestinal deterioration caused by low-dose DSS affects the development of systemic and intestinal hypersensitivity in a mouse model of cow’s milk allergy. Feeding mice 1–5% DSS polymers (molecular weight = 36,000–40,000 Da) in their drinking water for a few days induces a highly reproducible acute colitis characterized by body weight loss, diarrhea, rectal bleeding, and histological damage to the intestine [30]. In this study, we aimed to reflect early intestinal dysfunction by using 0.2% DSS in *β*-lg-sensitized mice to only slightly disorder the intestinal barrier’s function and approach the first events leading to mucosal damage and the development of allergy.

### 3.1. Sensitized β-lg Mice Respond to Low-Dose DSS with Reduced BW and Increased DAI Scores

**Figure 2:**
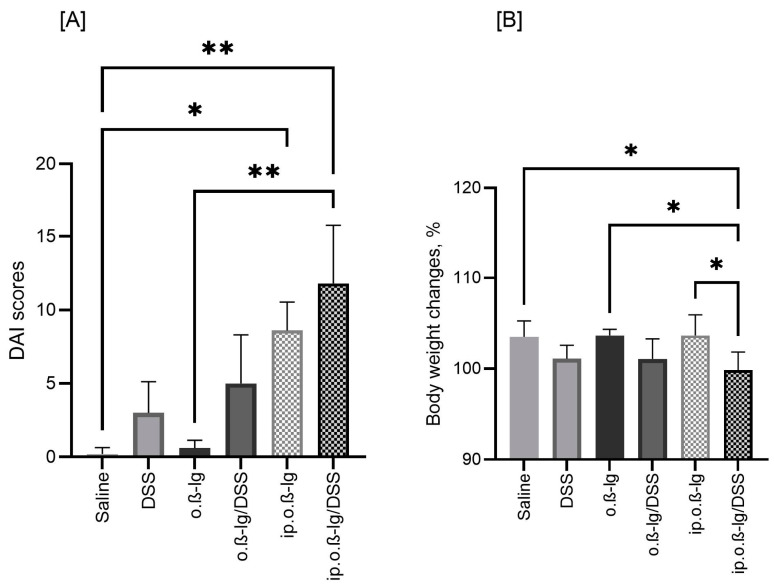
Changes in disease activity index (DAI) (**A**) and body weight (**B**) during challenge of mice with oral *β*-lg and DSS on days 21–31. Groups of mice: saline—healthy control group, saline administration at all stages; DSS—oral administration of DSS; o.*β*-lg—oral administration of *β*-lg; o.*β*-lg/DSS—oral administration of *β*-lg and DSS; ip.o.*β*-lg—intraperitoneal injection and oral administration of *β*-lg; ip.o.*β*-lg/DSS—intraperitoneal injection of *β*-lg and oral administration of *β*-lg and DSS. Data are expressed as group means ± SD. Means differ as follows: * at *p* ≤ 0.05, ** at *p ≤* 0.01.

Figure 2 illustrates the disease activity index (DAI) scores and body weight (BW) changes in the study groups during the challenge period when intraperitoneally sensitized mice were orally administered *β*-lg and DSS. DSS administration clearly reduced BW and increased DAI, although significant differences were observed only between the ip.o.*β*-lg/DSS and saline and o.*β*-lg groups (*p* < 0.05). The DAI of the ip.o.*β*-lg group also differed from that of the saline group (*p* < 0.05); however, the mean DAI score for ip.o.*β*-lg was almost one-third lower than that for ip.o.*β*-lg/DSS (Figure 2A). This is reflected in the differences in BW between these groups (ip.o.*β*-lg and ip.o.*β*-lg/DSS, *p* < 0.05; Figure 2B). The results show that the combination of intraperitoneal injection with oral administration of antigen in the presence of DSS increases the sensitivity of mice to the tested milk allergen.

### 3.2. No Expressed Effect of Low-Dose DSS on the Humoral Response of Mice

**Figure 3:**
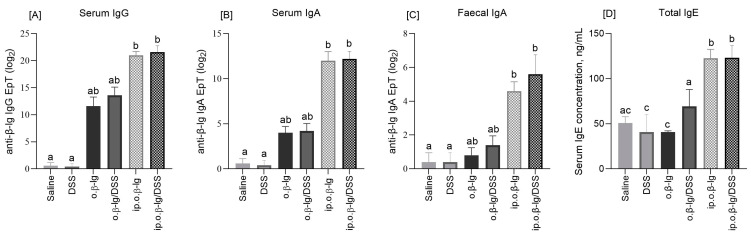
Terminal antibody endpoint titers (EpT; for IgG, IgA) and concentrations (for IgE) in blood serum (**A**,**B**,**D**) and fecal extracts (**C**) (secretory IgA). Groups of mice: saline—healthy control group, saline administration at all stages; DSS—oral administration of DSS; o.*β*-lg—oral administration of *β*-lg; o.*β*-lg/DSS—oral administration of *β*-lg and DSS; ip.o.*β*-lg—intraperitoneal injection and oral administration of *β*-lg; ip.o.*β*-lg/DSS—intraperitoneal injection of *β*-lg and oral administration of *β*-lg and DSS. Data are expressed as group means ± SD. Means with different superscripts (letters) are different at *p* ≤ 0.05.

Class G and A specific immunoglobulins are involved in the development of immune responses, including food allergy, both IgE-mediated and non-IgE-mediated, making them useful indicators of protein immunogenicity. We performed ELISA assays to compare the humoral response of the experimental groups (Figure 3). Treatment with DSS did not induce significant differences in the humoral response, either in serum or in feces. For anti-*β*-lg IgG and IgA, all groups responded to *β*-lg, but it was the intraperitoneally *β*-lg-injected groups of mice that were significantly different from the saline and DSS controls (*p* < 0.05). The total IgE increased by approximately 2.5-fold compared to the controls, but only in the ip.o.*β*-lg and ip.o.*β*-lg/DSS groups, demonstrating the efficiency of intraperitoneal immunization (*p* < 0.05). Fecal IgA concentration seems to be slightly increased by DSS, but these are not statistical differences, and they require further analysis. Also, a more precise analysis of the IgG antibody subgroups would perhaps clarify whether or not DSS at the dose tested affects the humoral response of *β*-lg-immunized mice. Chen et al. [11] compared different mouse sensitization models and reported the enhancing effect of DSS on OVA-specific IgE, IgG, IgG1, and IgG2 responses in OVA-sensitized mice with DSS-induced acute colitis, but their study lacks a comparison with the sensitized group without DSS administration. However, the results of Bouchikhi’s group [31] clearly showed that intestinal inflammation induced by DSS at a dose of 4% exacerbated the allergic sensitization and the humoral response of mice to egg white.

### 3.3. β-lg, Immunization Route and Low-Dose DSS affect T Cell Distribution in Inductive and Peripheral Tissues

**Figure 4:**
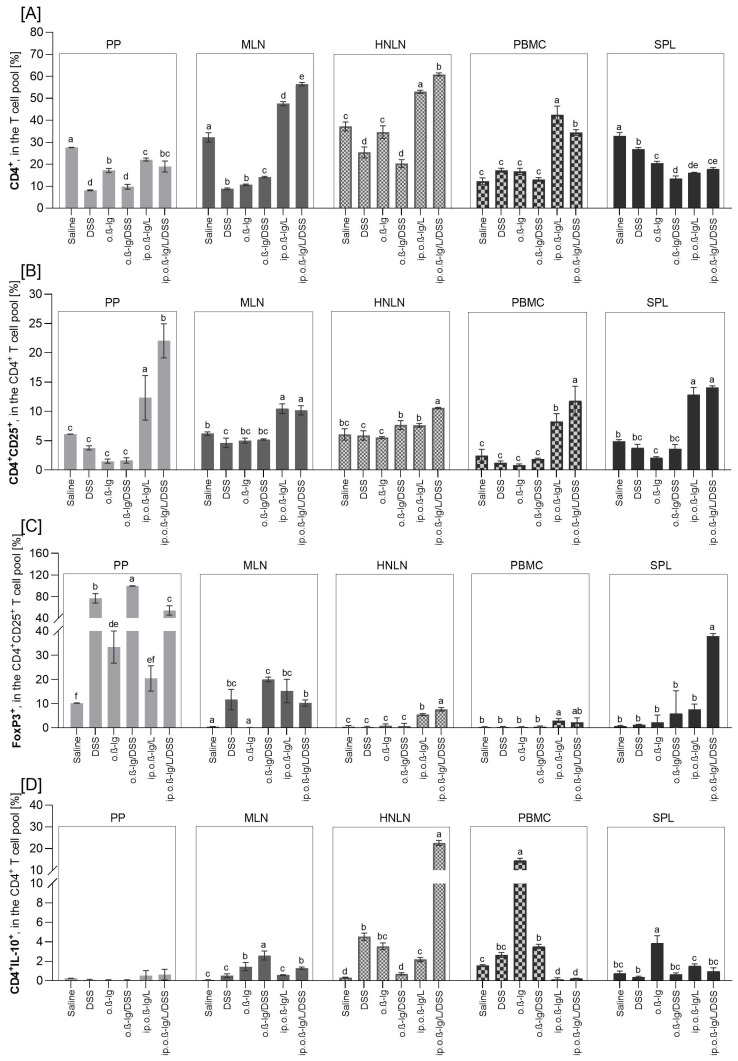
The distribution of CD4^+^ (**A**), CD4^+^CD25^+^ (**B**), CD4^+^CD25^+^Foxp3^+^ (**C**), and CD4^+^IL-10^+^ (**D**) T cells in Payer’s patches (PPs), mesenteric lymph nodes (MLNs), spleens (SPLs), head and neck lymph nodes (HNLNs), and peripheral blood mononuclear cells (PBMCs). Groups of mice: saline—healthy control group, saline administration at all stages; DSS—oral administration of DSS; o.*β*-lg—oral administration of *β*-lg; o.*β*-lg/DSS—oral administration of *β*-lg and DSS; ip.o.*β*-lg—intraperitoneal injection and oral administration of *β*-lg; ip.o.*β*-lg/DSS—intraperitoneal injection of *β*-lg and oral administration of *β*-lg and DSS. Data are expressed as the mean of the group ± SD. Means with different superscripts (letters) are different at *p* ≤ 0.05. Differences within the same tissue are shown.

**Figure 5:**
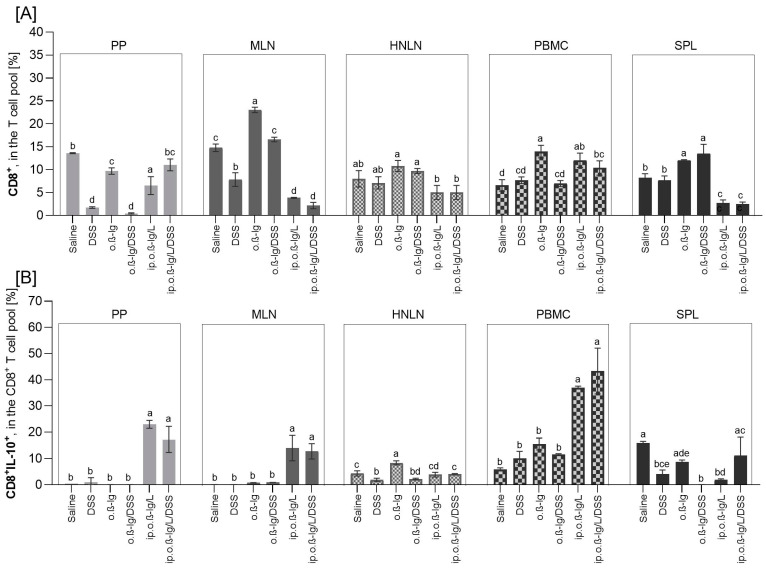
Distribution of CD8^+^ (**A**) and CD8^+^IL-10^+^ (**B**) T cells in Payer’s patches (PPs), mesenteric lymph nodes (MLNs), spleens (SPLs), head and neck lymph nodes (HNLNs), and peripheral blood mononuclear cells (PBMCs). Groups of mice: saline—healthy control group, saline administration at all stages; DSS—oral administration of DSS; o.*β*-lg—oral administration of *β*-lg; o.*β*-lg/DSS—oral administration of *β*-lg and DSS; ip.o.*β*-lg—intraperitoneal injection and oral administration of *β*-lg; ip.o.*β*-lg/DSS—intraperitoneal injection of *β*-lg and oral administration of *β*-lg and DSS. Data are expressed as the mean of the group ± SD. Means with different superscripts (letters) are different at *p* ≤ 0.05. Differences within the same tissue are shown.

T lymphocytes are crucial in the immune response to allergens. After initial contact with an antigen, naive mediator-driven CD4^+^ T cells can differentiate into subpopulations, such as Th1, Th2, Th17, and regulatory cells (Treg), and cause local activation of innate type 2 lymphocytes, followed by isotype switching of allergen-specific B cells into IgE-producing plasma cells that further induce allergic reactions. We analyzed the distribution of CD4^+^, CD4^+^CD25^+^, CD4^+^CD25^+^Foxp3^+^, CD8^+^, CD8^+^IL-10^+^, and CD4^+^IL-10^+^ T cells in Payer’s patches (PPs), mesenteric lymph nodes (MLNs), the spleen (SPL), head and neck lymph nodes (HNLNs), and peripheral blood mononuclear cells (PBMCs) (Figure 4 and Figure 5).

Oral administration of DSS and/or *β*-lg and intraperitoneal *β*-lg injection had an effect on the distribution of the T cells tested, and the effect was population- and tissue-dependent. DSS alone significantly reduced the percentage of CD4^+^ T cells in PP, MLN, SPL, and HNLN (Figure 4A), CD8^+^ cells in PP and MLN (Figure 5A), and CD8^+^IL-10^+^ cells in SPL and HNLN (Figure 5B), whereas it increased the population of FoxP3^+^ cells in PP and MLN (Figure 4C) and the population of CD4^+^IL-10^+^ cells in MLN and HNLN (Figure 4D) (*p* < 0.05 for DSS vs. saline and o.*β*-lg/DSS vs. o.*β*-lg groups). *β*-lg alone (o.*β*-lg group) decreased the percentage of CD4^+^ cells in PP, MLN, and SPL and CD4^+^CD25^+^ cells in SPL and CD8^+^ cells in PP, whereas it increased the proportion of FoxP3^+^ cells in PP, CD8^+^ in MLN and SPL, CD8^+^IL-10^+^ cells in HNLN, and CD4^+^IL-10^+^ cells in MLN, SPL, and HNLN (*p* < 0.05 vs. saline). The results showed that both *β*-lg and DSS affected T cell distribution.

Combined intraperitoneal *β*-lg injection and oral *β*-lg administration with or without DSS most significantly altered the profile of the T cells tested. At the site of first contact between the immune system and the ingested dietary antigens, i.e., in Peyer’s patches (clusters of lymph nodules in the mucosa and submucosa of the small intestine), the percentage of CD4^+^ cells decreased in the ip.o.*β*-lg and ip.o.*β*-lg/DSS groups, and CD8^+^ decreased in the ip.o.*β*-lg group, but CD4^+^CD25^+^ and CD8^+^IL-10^+^ cell populations increased in both groups, as did FoxP3^+^ cells in the ip.o.*β*-lg/DSS group (*p* < 0.5 vs. saline; Figure 4A–C and Figure 5A,B), with a higher percentage of the regulatory cells (CD4^+^CD25^+^ and FoxP3^+^CD4^+^CD25^+^) in the ip.o.*β*-lg/DSS group compared to the ip.o.*β*-lg group (*p* < 0.05). Thus, we observed the state of immune overstimulation and the body’s response in terms of reduced proliferation of CD4^+^ helper lymphocytes. Martino et al. [32] reported epigenetic dysregulation of T cell receptor complex signaling and poorer lymphoproliferative responses of naive CD4^+^ T cells in children with IgE-mediated food allergy.

**Figure 6:**
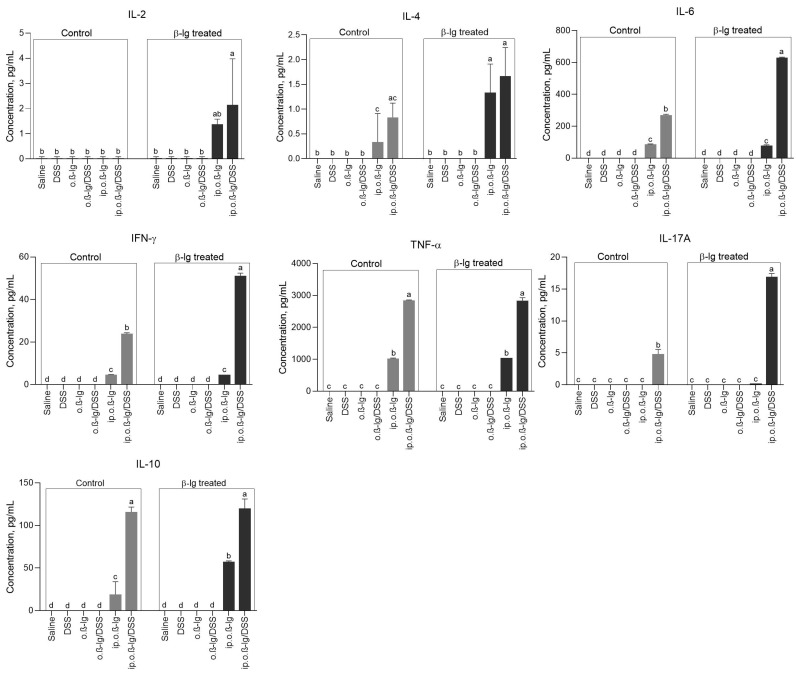
Cytokine levels in unstimulated (control) and *β*-lg-stimulated (*β*-lg-treated) mesenteric lymph node (MLN) cell cultures. Groups of mice: saline—healthy control group, saline administration at all stages; DSS—oral administration of DSS; o.*β*-lg—oral administration of *β*-lg; o.*β*-lg/DSS—oral administration of *β*-lg and DSS; ip.o.*β*-lg—intraperitoneal injection and oral administration of *β*-lg; ip.o.*β*-lg/DSS—intraperitoneal injection of *β*-lg and oral administration of *β*-lg and DSS. Data are expressed as the mean of the group ± SD. Means with different letters (12 groups were compared with each other) differ at *p* ≤ 0.05 (two-way ANOVA).

Foxp3-positive Treg lymphocytes (thymic and peripheral) are the subpopulation responsible for suppressing an overstimulated or autoreactive immune response, which is critical for maintaining immune tolerance at the tissue and systemic level [33]. They act by secreting inhibitory cytokines, suppressing membrane receptors action, and killing effector cells. However, it has also been shown that there are pro-inflammatory Treg cells, such as IL-4-producing FoxP3^+^ cells, IL-17-producing FoxP3^+^cells, and interferon-γ (IFN-γ)-producing FoxP3^+^ cells, which are strongly correlated with the severity of allergic diseases [34,35]. In this study, the CD4^+^CD25^+^ cells were generally highly increased in PPs, MLNs, and PBMCs compared to the control, but also in SPLs (*p* < 0.05; Figure 4B). We observed an increased number of these cells in HNLNs, but to a lesser extent and only in the ip.o.*β*-lg/DSS group (*p* < 0.05 vs. saline). DSS administered to mice injected intraperitoneally with *β*-lg increased the CD4^+^CD25^+^ cell population in the blood (*p* < 0.05 vs. ip.o.*β*-lg). Simultaneously, the number of FoxP3-positive CD4^+^CD25^+^ regulatory cells increased significantly compared to controls in all tissues analyzed except PP and SPL from the ip.o.*β*-lg group and PBMC from the ip.o.*β*-lg/DSS group (Figure 4C; *p* < 0.05 vs. saline). There were no significant differences in FoxP3^+^ regulatory cells in the MLN between the groups of mice injected intraperitoneally with *β*-lg and fed *β*-lg or *β*-lg with DSS. However, differences were observed in the HNLNs and SPLs, where DSS significantly stimulated FoxP3^+^ cells (*p* < 0.05 vs. ip.o.*β*-lg), indicating its significant effect on generating an immune response to the allergen tested. The balance between FoxP3 and GATA3 transcription factors ensures the suppressive function of thymic Treg cells [36]. We did not investigate this in the current study, so further studies are needed to clarify the direction of the response. In Th2-biased allergic diseases, the GATA-3 is the major factor regulating the transcription of Th2 lymphocytes. Overexpression of GATA-3 in tTreg cells induces the production of type 2 cytokines (IL-4, IL-5, IL-9, and IL-13). In turn, complete depletion of GATA-3 in mouse Treg cells resulted in thymic Treg cells that were defective in peripheral homeostasis and the suppressive function and that acquired a Th17 cell phenotype [37]. We found a significant increase in pro-inflammatory IL-17A secretion by MLN of the ip.o.*β*-lg/DSS group, a cytokine mainly secreted by activated Th17 cells (*p* < 0.05 vs. other groups; data presented in Section 3.4, Figure 6). FoxP3^+^ cells are generally increased in gastrointestinal inflammatory lesions, including Crohn’s disease [30,38]. The spleen is the largest hemolymphatic organ, where lymphocytes are activated by antigens from blood and differentiate into effector cells of the immune response, mainly humoral (after cooperation with T lymphocytes and dendritic cells). We observed a significant increase in Treg in the SPL of the ip.o.*β*-lg/DSS group, implying activity against an overstimulated or autoreactive immune response.

Interleukin-10 (IL-10) is a pleiotropic cytokine involved in the immunoregulation of inflammatory and antiallergic processes. There is ample evidence that IL-10 is involved in the generation of immunosuppression, while other evidence confirms the immunostimulatory properties of this cytokine [39,40]. Its action, like that of other cytokines, depends on the presence of its specific receptor, which has been shown to be expressed on the surface of APC cells, NK cells, and CD8^+^ and CD4^+^ T lymphocytes (including Tr1, Th2, and Th1), i.e., cells involved in the generation of the allergic response [39,41]. We found an increased number of CD4^+^IL-10^+^ T cells in the MLN of the ip.o.*β*-lg/DSS group and in the HNLN of the ip.o.*β*-lg and ip.o.*β*-lg/DSS groups, with a significantly higher number in the ip.o.*β*-lg/DSS group compared to the ip.o.*β*-lg group (Figure 4D; *p* < 0.05). In contrast, in PBMCs, we observed a decrease in this population in both groups (*p* < 0.05 vs. saline), which may be due to a fluctuation in the immune response or a weakening of its regulatory capacity.

CD8^+^ Tregs may also play an inhibitory role in the inflammatory response, although CD8^+^ T cells are uncommonly thought to be involved in IgE-mediated food allergy [42,43,44]. CD8 is a membrane glycoprotein that binds to the major histocompatibility complex class I (MHC class I) proteins. It is mainly found on cytotoxic T lymphocytes but also on dendritic cells, NK cells, and double-positive thymocytes. Our study showed that intraperitoneal immunization of mice and oral challenge with/without DSS had no effect or decreased the CD8^+^ T cell population in the tissues analyzed, except in PBMC, where an increase was observed (*p* < 0.05; Figure 5A). Tenorio et al. [45] found reduced subsets of CD8^+^ T cells in patients with IgE food allergy, while de Vos et al. [46] found reduced CD8^+^ T cells in the periphery of atopic toddlers with a history of wheeze, and the frequency of CD8^+^ T cells was negatively correlated with total serum IgE within the atopic group. We also found a decrease in peripheral CD8^+^ cells (MLN, HNLN) and an increase in serum antibodies (Figure 3 and Figure 5A). Regarding CD8^+^ T lymphocytes, an increased frequency of CD8^+^IL-10^+^ cells was found in the PPs, MLNs, and PBMCs of the ip.o.*β*-lg and ip.o.*β*-lg/DSS groups, with no effect of DSS (*p* < 0.05 vs. saline). This seems to indicate a potential role of CD8^+^ T cells in allergic sensitizing. Smith et al. [47] identified a regulatory loop in which IL-10 directly restricts CD8^+^ T cell activation and function through modification of cell surface glycosylation allowing the establishment of chronic viral infection. We noted a decrease in CD8^+^IL-10^+^ lymphocytes in the spleen of the ip.o.*β*-lg group, a hemolymphatic organ where lymphocytes are activated by antigens from the blood and differentiate into effector cells (*p* < 0.05 vs. saline).

### 3.4. Sensitized β-lg Mice Given Low-Dose DSS Produced More Pro-Inflammatory Cytokines

**Figure 7:**
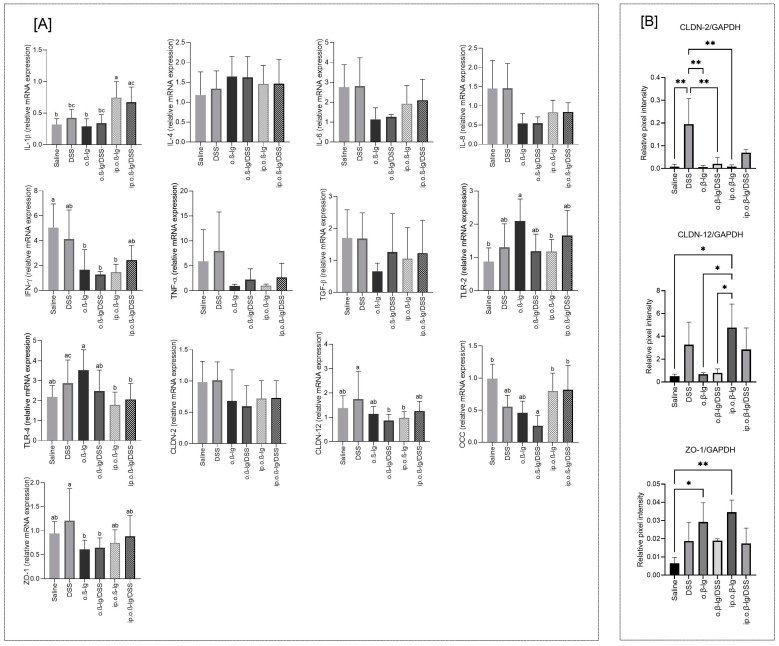
Effect of immunization method on the expression of interleukin-1beta (IL-1*β*), interleukin-4 (IL-4), interleukin-6 (IL-6), interleukin-8 (IL-8), interferon-gamma (IFN-γ), tumor necrosis factor alpha (TNF-α), toll-like receptor-2 (TLR-2), toll-like receptor-4 (TLR-4), claudin-2 (CLDN-2), claudin-12 (CLDN-12), occludin (OCC) and zonulin-1 (ZO-1) genes (**A**), and protein content (**B**) in the small intestinal tissue. Groups of mice: saline—healthy control group, saline administration at all stages; DSS—oral administration of DSS; o.*β*-lg—oral administration of *β*-lg; o.*β*-lg/DSS—oral administration of *β*-lg and DSS; ip.o.*β*-lg—intraperitoneal injection and oral administration of *β*-lg; ip.o.*β*-lg/DSS—intraperitoneal injection of *β*-lg and oral administration of *β*-lg and DSS. Data are expressed as the mean of the group ± SD. Means with different letters differ at *p* ≤ 0.05, with * differ at at *p* ≤ 0.05 and with ** differ at *p ≤* 0.01.

**Figure 8:**
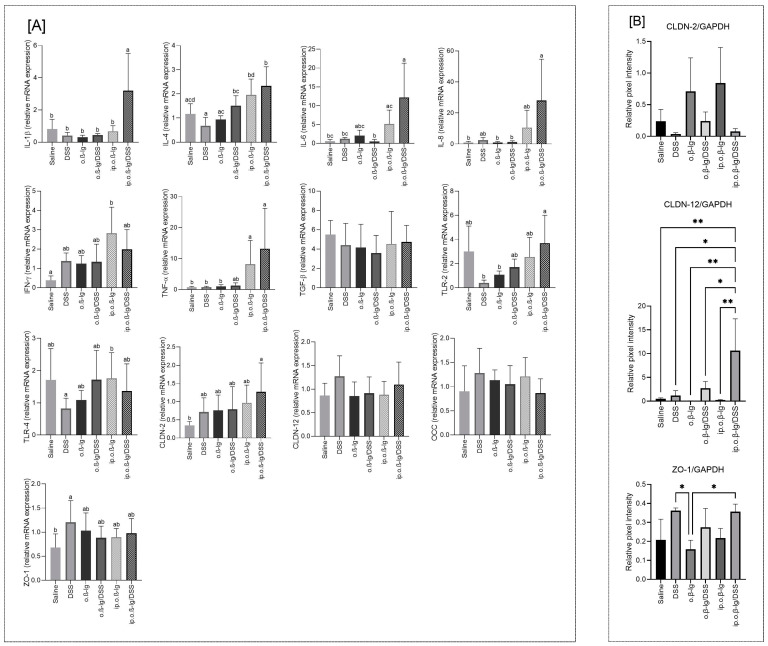
Effect of immunization method on the expression of interleukin-1beta (IL-1*β*), interleukin-4 (IL-4), interleukin-6 (IL-6), interleukin-8 (IL-8), interferon-gamma (IFN-γ), tumor necrosis factor alpha (TNF-α), toll-like receptor-2 (TLR-2), toll-like receptor-4 (TLR-4), claudin-2 (CLDN-2), claudin-12 (CLDN-12), occludin (OCC) and zonulin-1 (ZO-1) genes (**A**), and protein content (**B**) in the colonic tissue. Groups of mice: saline—healthy control group, saline administration at all stages; DSS—oral administration of DSS; o.*β*-lg—oral administration of *β*-lg; o.*β*-lg/DSS—oral administration of *β*-lg and DSS; ip.o.*β*-lg—intraperitoneal injection and oral administration of *β*-lg; ip.o.*β*-lg/DSS—intraperitoneal injection of *β*-lg and oral administration of *β*-lg and DSS. Data are expressed as the mean of the group ± SD. Means with different letters differ at *p* ≤ 0.05, with * differ at at *p* ≤ 0.05 and with ** differ at *p ≤* 0.01.

Provoking the intestine of sensitized mice with inflammatory DSS strongly altered cytokine secretion. Concentrations of cytokines tested (IL-2, IL-4, IL-6, IFN-γ, TNF-α, IL-17A, and IL-10) in the analyzed tissue (MLN) in the saline, DSS, o. *β*-lg, and o. *β*-lg/DSS groups and for IL-17A in the ip.o.*β*-lg group were below detection level but significantly increased in the ip.o.*β*-lg and/or ip.o.*β*-lg/DSS groups (*p* < 0.05 vs. saline; Figure 6). Furthermore, significant differences were found between ip.o.*β*-lg and ip.o.*β*-lg/DSS groups, indicating a strong stimulatory effect of DSS on the secretion of cytokines, such as IL-6, IFN-λ, TNF-α, IL-17A, and IL-10. With the exception of TNF-α, for which the results were comparable, antigen stimulation of cultured MLN cells of sensitized mice increased cytokine secretion, but mainly by ip.o.*β*-lg/DSS-derived cells, revealing a significant effect of DSS on mouse immunization and ongoing inflammation (*p* < 0.05). This correlates with the generally higher T-lymphocyte activity in this group and may be an attempt to suppress an autoreactive immune system response. However, according to Xu et al. [48], pro-inflammatory IL-6 leads to the loss of FoxP3 expression in tTreg cell, affecting the stability of tTreg cells and resulting in the conversion of tTreg cells to Th17 cells. TGF-*β* controls proliferation and differentiation in most cell types and is a critical differentiation factor for the generation of Treg cells [48,49,50]. We did not find an increase in the expression of genes encoding TGF-*β* in the intestinal tissue of sensitized mice (Figure 7 and Figure 8). However, we did observe an increase in *IL-6* gene expression in the colonic tissue of the ip.o.*β*-lg/DSS group and an increase in its concentration in the blood of the sensitized groups of mice, especially those receiving DSS, which may inhibit TGF-*β*-induced CD4^+^CD25^+^FoxP3^+^ production, as IL-6 and TGF-*β* together can only increase the induction and differentiation of pathogenic Th17 lymphocytes from naïve T cells [51]. Furthermore, there are indications that Treg can induce CD4^+^CD25^+^FoxP3^+^ cells or are self-induced to become Th17 cells in the absence of exogenous TGF-*β* [48]. A subset of IL-17-producing Th17 cells plays a crucial role in the induction of autoimmune tissue injury [52]. We found a significant increase in the secretion of pro-inflammatory IL-17A by MLN from the ip.o.*β*-lg/DSS group, which received DSS and had the highest DAI scores (Figure 2 and Figure 6; *p* < 0.05 vs. saline/or other groups). Similarly, the group showed activity of IL-2, which is produced and secreted by antigen-stimulated T lymphocytes and plays an important role in regulating the balance between Th17 and FoxP3 regulatory cells (Figure 6).

### 3.5. Gut-Tissue-Dependent Effect of β-lg and Low-Dose DSS on Gene Expression of Regulatory Molecules

Increased intestinal permeability allows allergens to penetrate the intestinal barrier and stimulate the submucosal immune system, while the cytokines and inflammatory mediators released increase epithelial barrier degradation and dysfunction [29,53]. We examined the gene expression of cytokines with pro-inflammatory activity (IL-1*β*, IL-4, IL-6, IL-8, IFN-λ, and TNF-α), transforming growth factor beta (TGF-*β*), toll-like receptors (TLR-2 and TLR-4), and tight junction (TJ) components, such as claudins (CLDN-2 and CLDN-12), occludin (OCC), and zonula occludens-1 (ZO-1), in the small and large intestines of the tested groups of mice. DSS administration had no effect on the expression of most of these genes in the small intestine (Figure 7A). However, we observed an increased expression of IL-1*β* in intraperitoneally immunized mice (*p* < 0.05 vs. saline; Figure 7A). IL-1*β* is involved in the modulation of autoimmune inflammation in the absence of additional signals and in combination with IL-23-induced expression of IL-17, IL-21, and IL-22 by γδ T cells [54]. IL-1*β* can also promote intestinal inflammation by interfering with the TJ barrier and allowing increased penetration of antigens from the intestinal lumen [55,56]. We observed a decrease in the expression of IL-6, IL-8, IFN-γ, and TNFα encoding genes in the *β*-lg-treated mice, though without statistical significance, except for IFN-λ and o.*β*-lg, o.*β*-lg/DSS, and ip.o.*β*-lg groups, which may be due to downregulation. The expression of both Toll-like receptors tested increased only in the o.*β*-lg group, possibly due to an altered microbiota balance (*p* < 0.05 vs. saline for TLR-2; Figure 7A). As for the TJ components encoding genes in small intestinal tissue, we did not observe any obvious differences, but the expression of genes encoding CLDN-12 and ZO-1 was significantly higher in the DSS group compared to the o.*β*-lg and o.*β*-lg/DSS groups, and OCC was significantly lower in the o.*β*-lg/DSS group compared to the saline group (*p* < 0.05; Figure 7A). Also, immunoblotting revealed differences when analyzing CLDN-2, CLDN-12, and ZO-1 (Figure 7B). DSS seemed to increase the production of both analyzed claudins. In turn, ZO-1 increased in all experimental groups, but the effect was significant only in the o.*β*-lg and ip.o.*β*-lg groups (*p* < 0.05 vs. saline).

A more pronounced influence of the studied factors on the analyzed genes and proteins was observed in the colonic tissue, the target site of DSS activity (Figure 8A,B). We observed a significant increase in the expression of most of the pro-inflammatory cytokine genes analyzed in the ip.o.*β*-lg/DSS group (*p* < 0.05 vs. saline; Figure 8A). The expression of *IFN-λ* and *TNF-α* genes also increased in the ip.o.*β*-lg group (*p* < 0.05 vs. saline). Both of these cytokines are strongly associated with allergy [57,58,59]. Because the relative mRNA expression was generally higher in the ip.o.*β*-lg/DSS group compared to the ip.o.*β*-lg group, with the exception of IFN-γ, it appears that the dose of DSS used exerts a significant pro-inflammatory effect on the colonic tissue of *β*-lg-sensitized mice at a site where administering this agent at high doses causes colitis.

We did not observe a significant effect of DSS on colonic TLRs, although intraperitoneal *β*-lg injection combined with oral *β*-lg and DSS seems to enhance TLR-2 gene expression (*p* < 0.05 vs. DSS and o.*β*-lg). Similarly, an increasing trend was observed in the experimental groups for CLDN-2 (Figure 8A). However, only the ip.o.*β*-lg/DSS and saline groups were statistically different (*p* < 0.05). Claudins are a family of proteins that, together with occludin, are the major components of tight junctions, which form the paracellular barrier that controls the flow of molecules in the intracellular space between epithelial cells [60,61,62]. Their mode of action is not yet fully understood. According to Lameris et al. [63], they show distinct expression patterns throughout the gastrointestinal tract, and claudin-12 mRNA is significantly upregulated in the ileum of Crohn’s disease patients, whereas claudin-2 mRNA is significantly reduced in the sigmoid colon compared to healthy controls. Our immunoblotting results showed that DSS decreased CLDN-2 and increased CLDN-12 production (*p* < 0.05 vs. saline for CLDN-12; Figure 8B). Zonula occludens-1 (ZO-1) is a peripheral membrane protein, a scaffold protein that cross-links and anchors TJ strand proteins. The role of zonulin-related proteins and ZO-1 in intestinal permeability has been implicated in a number of diseases, including celiac disease, type 1 diabetes, and type 2 diabetes [64,65]. According to Kuo et al. [66] ZO-1 primarily promotes mucosal repair, and its loss contributes to defective mucosal healing in inflammatory bowel disease. We observed an increase in ZO-1 mRNA expression in the colonic tissue of the DSS group, presumably in response to the unsealing/detrimental activity of DSS on the colonic epithelium (*p* < 0.05 vs. saline; Figure 8A). This effect appears to be supported by Western blot data, where we observed increased levels of ZO-1 concentration in colonic tissue in the DSS-treated groups (Figure 8B). Thus, this study showed that DSS at low doses impairs the intestinal barrier.

### 3.6. Sensitizing Mice with β-lg in the Presence of Low-Dose DSS Does Not Cause Major Shifts in Total Bacterial Counts, but It Does Affect the Microbiota Balance and the Profile of Volatile Fatty Acids

The microbiota has been shown to have a significant impact on the development of food allergies [8]. Children with allergies have a less diverse microbiota, including fewer *Bacteroidetes*, *Bifidobacterium,* and *Lactobacillus*, and more clostridia [67,68]. We analyzed the total number of bacteria (TNB) and the populations of *Lactobacillus* and *Bifidobacterium* in the cecal contents of the studied groups of mice (Table 2). We found no differences in the TNB or in the abundance of the *Bifidobacterium* populations, but the *Lactobacillus* populations differed significantly between the groups tested (Table 2). The DSS, oral *β*-lg, and ip.o.*β*-lg/DSS groups had significantly fewer lactobacilli than the saline group, and the ip.o.*β*-lg/DSS group also had fewer than the ip.o.*β*-lg group (*p* < 0.05), implying the impact of DSS on the gut ecosystem, including the microbiota and associated inflammatory and allergic processes.

Analysis of the short-chain fatty acids (SCFAs) showed differences between the groups of mice studied in the concentrations of some of the acids analyzed (Table 3). Compared to saline, the total SCFA decreased in the o.*β*-lg/DSS and ip.o.*β*-lg groups, and acetic and valeric acids decreased in the ip.o.*β*-lg group (*p* < 0.05). Although we observed an upward trend in acid concentrations in the ip.o.*β*-lg/DSS group, only the valeric acid content and the sum of putrefactive short-chain fatty acid content (total PSCFA, sum of isovaleric, valeric, and isobutyric acids; Table 3) differed from the saline control group (*p* < 0.05). Except for butyrate and isovalerate, there were differences in the SCFA analyzed between the ip.o.*β* and ip.o.*β*/DSS groups (*p* < 0.05). The ip.o.*β* /DSS group generally had higher levels of SCFA, including putrefactive ones. The observed microbiota activity and SCFA production in the ip.o.*β*-lg/DSS group may be due to disorders of gut integrity and defense mechanisms induced by DSS and the resulting increased activity of selected groups of bacteria [30].

## 4. Conclusions

The pathogenesis of allergy is extremely complex, involving interactions between numerous factors and mechanisms. This study is the first to provide a detailed characterization of the immune response to milk allergen in a mouse model in the presence of low-dose DSS as an inducer of early intestinal dysfunction. We have shown that low-dose DSS enhances the efficiency of immunization of mice with bovine *β*-lg, affecting DAI, BW, T cell induction in peripheral tissues, activation of immune mediators, and microbiota balance and activity. Low-dose DSS appears to be suitable for studying the physiology of the intestinal barrier and changes that may provide insight into the first events leading to mucosal dysfunction in the early stages of food allergy development. Given that a hypersensitive response to milk constituents may be a humoral response, a cellular response, or an indirect response involving both cells and IgE antibodies, and taking into account the numerous interactions between different types of cells and processes of the immune system, it is not possible at present to say whether the described disorders underline the development of food allergy or whether they are only part of its complex pathomechanism. Further research to evaluate the possible pathways of food allergy development and to fine-tune the DSS dose for the model is warranted. Nevertheless, the presented experimental design appears to be a good addition to current scientific research, and the results obtained have important implications for understanding immune dysregulation in food hypersensitivity. The results can be used in preclinical studies to investigate impaired protein digestion and intestinal mucosal barrier function, including the breakdown of tolerance mechanisms.

## Figures and Tables

**Table 2:** Bacterial counts in the cecal contents (log cells/g ± SD) ^1,2^.

	Saline	DSS	o.*β*-lg	o.*β*-lg/DSS	ip.o.*β*-lg	ip.o.*β*-lg/DSS
TNB ^3^	10.44 ± 0.23	10.34 ± 0.12	10.63 ± 0.20	10.26 ± 0.25	10.24 ± 0.04	10.35 ± 0.04
*Lactobacillus* spp.	7.78 ± 0.19 ^b^	7.42 ± 0.03 ^cd^	7.06 ± 0.12 ^a^	7.62 ± 0.08 ^bd^	7.64 ± 0.02 ^bd^	7.11 ± 0.13 ^ac^
*Bifidobacterium* spp.	6.08 ± 0.49	6.08 ± 0.13	5.93 ± 0.06	6.04 ± 0.18	5.76 ± 0.32	6.00 ± 0.12

^1^ Groups of mice: saline—healthy control group, saline administration at all stages; DSS—oral administration of DSS; o.*β*-lg—oral administration of *β*-lg; o.*β*-lg/DSS—oral administration of *β*-lg and DSS; ip.o.*β*-lg—intraperitoneal injection and oral administration of *β*-lg; ip.o.*β*-lg/DSS—intraperitoneal injection of *β*-lg and oral administration of *β*-lg and DSS. ^2^ Data are expressed as the mean of the group ± SD. Means with different letters differ at *p* ≤ 0.05. ^3^ TNB—total number of bacteria.

**Table 3:** Concentration and profile of short-chain fatty acids (SCFAs) in the cecal digesta of mice (mean ± SD) ^1,2^.

	Saline	DSS	o.*β*-lg	o.*β*-lg/DSS	ip.o.*β*-lg	ip.o.*β*-lg/DSS
SCFA, µmol/g of digesta						
Acetic acid	60.20 ± 10.21 ^bd^	64.17 ± 7.16 ^be^	54.26 ± 8.55 ^cde^	47.32 ± 1.30 ^ad^	35.72 ± 1.93 ^a^	71.22 ± 8.34 ^b^
Propionic acid	10.71 ± 5.53 ^ab^	13.19 ± 4.84 ^ac^	7.54 ± 1.82 ^bc^	9.20 ± 1.18 ^ab^	5.27 ± 1.87 ^b^	15.79 ± 3.04 ^a^
Butyric acid	21.27 ± 8.72	15.89 ± 3.24	23.78 ± 2.44	13.41 ± 0.55	12.70 ± 2.48	23.27 ± 8.65
Valeric acid	0.57 ± 0.04 ^cd^	0.53 ± 0.04 ^cd^	0.68 ± 0.11 ^ac^	0.49 ± 0.06 ^bd^	0.35 ± 0.02 ^b^	0.77 ± 0.10 ^a^
Isovaleric acid	0.14 ± 0.03 ^ab^	0.14 ± 0.05 ^ab^	0.22 ± 0.03 ^b^	0.23 ± 0.06 ^b^	0.12 ± 0.01 ^a^	0.20 ± 0.06 ^ab^
Isobutyric acid	0.47 ± 0.04 ^ab^	0.57 ± 0.10 ^ab^	0.43 ± 0.16 ^ab^	0.51 ± 0.09 ^ab^	0.35 ± 0.07 ^a^	0.62 ± 0.20 ^b^
Total PSCFA ^3^	1.17 ± 0.06 ^c^	1.24 ± 0.18 ^c^	1.33 ± 0.19 ^bcd^	1.22 ± 0.18 ^c^	0.81 ± 0.08 ^a^	1.59 ± 0.07 ^bd^
Total SCFA	93.36 ± 9.86 ^bd^	94.48 ± 8.50 ^bd^	86.91 ± 12.86 ^cd^	71.15 ± 1.88 ^ac^	54.50 ± 4.52 ^a^	111.87 ± 8.04 ^b^
Profile, % of total SCFA						
Acetic acid	64.22 ± 5.02	67.83 ± 1.63	62.36 ± 0.83	66.52 ± 1.72	65.67 ± 2.20	63.60 ± 5.31
Propionic acid	11.30 ± 4.94	13.71 ± 4.26	8.58 ± 0.93	12.91 ± 1.32	9.61 ± 2.94	14.27 ± 3.39
Butyric acid	23.22 ± 9.78	17.14 ± 5.09	27.52 ± 1.48	18.85 ± 0.75	23.22 ± 3.48	20.70 ± 7.27
Total PSCFA	1.26 ± 0.12 ^ab^	1.32 ± 0.22 ^a^	1.54 ± 0.21 ^b^	1.73 ± 0.29 ^ab^	1.50 ± 0.11 ^ab^	1.43 ± 0.12 ^ab^

^1^ Groups of mice: saline—healthy control group, saline administration at all stages; DSS—oral administration of DSS; o.*β*-lg—oral administration of *β*-lg; o.*β*-lg/DSS—oral administration of *β*-lg and DSS; ip.o.*β*-lg—intraperitoneal injection and oral administration of *β*-lg; ip.o.*β*-lg/DSS—intraperitoneal injection of *β*-lg and oral administration of *β*-lg and DSS. ^2^ Data are expressed as the mean of the group ± SD. Means with different letters differ at *p* ≤ 0.05. ^3^ PSCFA—putrefactive short-chain fatty acid (the sum of valeric, isovaleric, and isobutyric acids).

## Data Availability

All data are presented in this study, and details may be made available upon request from the corresponding author.
